# Book Reviews

**Published:** 1895-12

**Authors:** 


I. The Physicians' Visiting List (Lindsay & Blakiston) for 1896. Forty-fifth year of its publication. Sold by all booksellers and druggists. Philadelphia: P. Blakiston, Son & Co., 1012 Walnut street.

II. The Medical Record Visiting List and Physicians' Diary for 1896. New Revised Edition. With Calendar, Tables of Doses, Tables of Equivalents, Directions for Emergencies, Antisepsis, Disinfection, Special Memoranda, Cash Account, etc., etc., thirty and sixty patients per week, bound in black or red morocco leather with flap, $1.25 and $1.50. Circular on application. New York: William Wood & Company, Publishers.

III. The Medical News Visiting List for 1896. Weekly (dated, for thirty patients); Monthly (undated, for 120 patients per month); Perpetual (undated, for thirty patients weekly per year); and Perpetual (undated, for sixty patients weekly per year). The first three styles contain thirty-two pages of data and 160 pages of blanks. The sixty-page Perpetual consists of 256 pages of blanks. Each style in one wallet-shaped book, with pocket, pencil and rubber. Seal grain leather, $1.25. Philadelphia; Lea Brothers & Co. 1895.

I. The Lindsay & Blakiston visiting list, so well known to every practising physician, appears with its usual promptness and presents several improvements in the edition for 1896. More space has been allowed for writing the names and to the memoranda page; a column has been added for the amount of the weekly visits and a column for the leger page. To do this without increasing the bulk or price, the reading matter and memoranda pages have been rearranged and simplified. The lists for seventy-five patients and 100 patients will also have special memoranda page as above, and hereafter will come in two volumes only, dated January to June and July to December. While this makes a book better suited to the pocket, the chief advantage is that it does away with the risk of losing the accounts of a whole year should the book be mislaid. The publishers announce that before making these changes they have personally consulted a number of physicians who have used the book for many years, and have taken into consideration many suggestions made in letters from all parts of the country.

This visiting list is the oldest in the market, but it keeps itself abreast of professional progress. The prices range from $1.00 to $2.25, according to size.

II. The Medical Record Visiting List for 1896 is fully up to its old standard of excellence. There are thirty closely printed pages, preceding the visiting list proper, that contain the tables and other items noted in the title above. The paper is fine, the arrangement for recording visits and other memoranda is excellent and the binding, which is the best morocco, soft and flexible, is second to none. It is published in two sizes, for thirty and sixty patients a week, and either dated for 1896 or without dates, to suit the requirements of the physician. It is difficult to see how this visiting list could be improved upon.

III. The Medical News Visiting List for 1896 still maintains its claim to popular favor as one of the best in the field. This edition has been thoroughly revised and brought forward to the present period in all of its appointments. The thirty-two pages of text preceding the daily record contains the usual tables of reference that are so valuable to busy physicians. The visiting list proper and other memoranda pages are classified in most excellent manner. The mechanical execution of the book is unsurpassed in paper, binding and cover. A ready reference thumb-letter index is a time-saving device that repays for its small additional cost, 25 cents. Taken altogether, it is one of the most satisfactory visiting lists extant.

Twentieth Century Practice. An International Encyclopedia of Modern Medical Science. By leading authorities of Europe and America. Edited by Thomas L. Stedman, M. D., New York City. In twenty volumes. Volume IV. Diseases of the Vascular System and Thyroid Gland. New York: William Wood & Company. 1895.

The first section of this volume, consisting of 454 pages, is devoted to the consideration of diseases of the heart and pericardium, and is written by James T. Whittaker, of Cincinnati. The first sixty pages of this monograph are taken up by diseases of the pericardium, while the remainder, nearly 400 pages, treat of diseases of the heart itself and the endocardium.

This is by far the most exhaustive treatment that the heart has ever received in literature, and reflects an everlasting credit upon the distinguished author. Whittaker is as interesting in his pathology as he is in therapeutics, which is alike a compliment to his rhetoric and to his knowledge of the subject. It is as pleasant to read his description of aortic insufficiency as it is to trace Sherlock Holmes through the mazes of an intricate criminal investigation. Whoever should follow these lines closely and study them carefully will learn something about the heart that he never knew before.

In the next section, comprising about 100 pages, we find diseases of the blood-vessels considered at the hands of Arthur Ernest Sansom, of London, whose reputation is fast growing into conspicuous eminence. This is a subject that has received less attention heretofore than its importance would seem to merit. A considerable portion of this section is devoted to the study of chronic aortitis—atheroma—which is for the first time treated in literature with anything like exhaustive precision. Aortic aneurism and arteriosclerosis are admirably set forth by this accomplished author. The third section, consisting of about forty-five pages, discourses upon diseases of the lymphatic vessels, and is from the pen of Bertrand Dawson, of London. Here again may be found much of interest not elsewhere available.

The final section, on diseases of the thyroid gland, including myxedema, cretinism, exophthalmic goiter, goiter and inflammation and neoplasms, is prepared by George R. Murray, of Newcastle-on-Tyne. This author introduced the thyroid gland treatment for myxedema and cretinism, which has made him so famous. In this monograph he enters very fully into the history and application of the thyroid treatment and presents the subject in systematic form. Heretofore the searcher could only find scattering journal articles by the originator of the method, but now he will be able to examine an exhaustive commentary written by Dr. Murray himself and placed within the covers of this volume, which, in respect to the importance of the subjects treated or the justly celebrated reputation of the authors, is not second to any that have preceded it in these series.

---

Annual of the Universal Medical Sciences. A Yearly Report of the Progress of the General Sanitary Sciences throughout the World. Edited by Charles E. Sajous, M. D., and seventy associate editors, assisted by over 200 corresponding editors, collaborators and correspondents. Illustrated with chromo-lithographs, engravings and maps. Five volumes. The F. A. Davis Company, Publishers, Philadelphia, New York, Chicago. London: F. J. Rebman. Australian Agency: Melbourne, Victoria, 1895.

The eighth edition of this important work has been delayed somewhat in presenting itself, but it is none the less welcome for all that. It still maintains its reputation as one of the best condensations of the medical literature of the year that has yet been presented for the favor of the medical profession. The slight delay in its appearance, due to causes entirely beyond the control of the editor, does not detract from its value as a work of reference. The fact is, year by year, its worth increases with reference to its early issues, because it keeps the literature of bygone years, always more difficult to reach than that of the present, within easy command.

There have been some changes in the editorial staff, but in the main it stands as heretofore and the general plan of the work has not been changed. The editor, in his preface, pays a just tribute to the memory of Dujardin-Beaumetz, which is eloquent in diction and finished in rhetoric.

We here and now affirm an opinion heretofore expressed that it would be difficult for the teacher, editor, writer or progressive physician to dispense with this comprehensive and most useful work. It would be missed more than any other of its kind should it discontinue its annual visits.

Transactions of the Medical Society of the State of New York for the Year 1895. Edited by Frederic C. Curtis, M. D., secretary. Published by the Society. William J. Dornan, Printer. 1895.

The annual volume showing the work of this society presents itself in excellent form. The financial status of the society was never better than at present, nor were its transactions ever published in a more attractive or substantial manner. The president, Dr. George Henry Fox, departed from the usual custom and read his address on the afternoon session of the second day, instead of in the evening at the capitol. The subject of his address, Credulity and scepticism in modern medicine, proved interesting as well as instructive. The usual number of papers and discussions follow and then the obituaries. One of the most useful features of these volumes is the publication of the lists of members, honorary, permanent, delegate and those of county societies.

This society would do itself injustice if it changed the form and method of the publication of its transactions. A standard octavo is the proper size for a library book, and in its present form this volume is neither too cheap nor too expensive. There should be no further attempt to cheapen the publication in price at the expense of quality.

A TEXT-BOOK ON PRACTICAL THERAPEUTICS, with Especial Reference to the Application of Remedial Measures to Disease and Their Employment upon a Rational Basis. By HOBART AMORY HARE, M. D., Professor of Therapeutics and Materia Medica in the Jefferson Medical College of Philadelphia. With special chapters by Drs. G. E. de Schweinitz, Edward Martin and Barton C. Hirst. New (fifth) edition, thoroughly revised. In one octavo volume of 740 pages. Cloth, $3.75; leather, $4.75. Philadelphia: Lea Brothers & Co., Publishers. 1895.

Not before in our experience have we been called upon to notice a fifth edition of a book within five years of its first appearance. This fact not only bespeaks the popularity of this work, but it is a strong testimonial to its value as a text-book; for no work on this subject without special merit could present such a history. In the present edition some changes have been made in the way of revision, suggestions, additions and, in some instances, articles have been rewritten. The antitoxin treatment of diphtheria is briefly discussed and the author gives a résumé of our present knowledge on the subject.

One of the particular reasons for the popularity of this work consists of the close relationship between the two departments, therapeutics and materia medica. Hare has closely interwoven them and yet treated them in separate and distinctive sections. We apprehend the demand for this useful treatise will continue and that the present edition will become speedily exhausted. In the ever-changing nature of many portions of the subject it is, perhaps, fortunate that it should be so.

A Manual of Obstetrics. By A. F. A. King, M. D., Professor of Obstetrics and Diseases of Women in the Medical Department of the Columbian University, Washington, D. C., and in the University of Vermont, etc. New (sixth) edition. In one 12mo volume of 532 pages, with 221 illustrations, Cloth, $2.50. Philadelphia: Lea Brothers & Co. 1895.

This book is well and favorably known to the medical profession. That it has progressed to a sixth edition since it first appeared, in 1882, is manifest evidence of its value and ought to afford gratification to its author, which no doubt it does. In the present edition some previous errors have been corrected, and a number of additions and modifications made. Seventy-one new illustrations have been added, borrowed, for the most part, from the treatises of Parvin, Hirst, Leishman, Playfair and Galabin. The publishers have made a handsome volume.

---

LECTURES ON APPENDICITIS AND NOTES ON OTHER SUBJECTS. By ROBERT T. MORRIS, A. M., M. D., Fellow of the New York Academy of Medicine, American Association of Obstetricians and Gynecologists, etc., etc. Octavo, pp. xviii.—163. With illustrations by HENRY MACDONALD, M. D. New York: G. P. Putnam's Sons, 27 West Twenty-third street. 1895.

This author has a special knowledge of the subject treated of in this book. He has a peculiar aptitude for imparting a clear understanding of his subject to his audience—listeners or readers. He has something to say, says it and stops. When a man writes a sentence like the following we naturally nestle close to him during his discourse, written or oral. Dr. Morris says in his preface: "In the matter of operative procedures I have due respect for methods which are different from my own, believing that in the art of surgery every surgeon is a law unto himself and that he knows the factors of his own success."

The first chapter of the book is devoted to the consideration of surgical cleanliness under the title, preparation of surgeon and patient. It is the clearest and most concise statement of the case we have ever seen, and the wayfaring man, though a fool, could not fail to understand it. To make the field of operation and the surgeon's hands surgically clean is not a difficult procedure in fact; it has only been made so by the unnecessarily complicated rules of surgical diletanti and devotees of everything that is novel.

The author's anatomical description is brief and clear, and when he approaches the subject proper he rises to the occasion with a sublimity that is striking as well as instructive. He first describes appendicitis and then its surgical treatment. His technique is beyond criticism. He describes it step by step, dividing the operation by his famous inch and a half incision into seven steps. Each step is minutely detailed and a reason given for each section or subdivision thereof.

Morris's results are surprisingly good. He publishes a table of 100 cases—his first series—with eight deaths. His later series show even fewer fatal cases.

Taken altogether, this is one of the most instructive monographs upon appendicitis that we have had the privilege of examining. About one-half of this book is devoted to the consideration of other subjects, but its chief interest centers around appendicitis. Yet it must be confessed there are many practical surgical hints in the notes, that comprise about one-half the volume.

The book is printed on plate paper and is handsomely illustrated. It should be found on the table of every practical surgeon.

